# Rab23 promotes squamous cell carcinoma cell migration and invasion via integrin β1/Rac1 pathway

**DOI:** 10.18632/oncotarget.6701

**Published:** 2015-12-21

**Authors:** Qiang Jian, Ye Miao, Li Tang, Min Huang, Yi Yang, Wei Ba, Yali Liu, Sumin Chi, Chengxin Li

**Affiliations:** ^1^ Department of Dermatology, Xijing Hospital, Fourth Military Medical University, Xi'an, Shaanxi Province, China; ^2^ Department of Dermatology, Chinese People's Liberation Army General Hospital, Beijing, China; ^3^ Department of Physiology, Fourth Military Medical University, Xi'an, Shaanxi Province, China

**Keywords:** Rab23, squamous cell carcinoma, invasion, integrin β1, Rac1

## Abstract

Rab23 was a member of Ras-related small GTPase family, which played a key role in the regulation of Shh signaling pathway. However, the function and regulatory mechanism of Rab23 in cutaneous squamous cell carcinoma was unknown. In this study, we found that the expression level of Rab23 was higher in moderately to poorly tumor differentiation tissue and non-exposed positions, and no statistically significant difference showed in Rab23 expression according to trauma/chronic disease, location on lips/ears, tumor size, gender, or age. Interestingly, we found that Rab23 RNAi suppressed cell invasion and Rab23 overexpression promoted cell invasion depended on GTP-bound form of Rab23. Inhibition of Rac1 activity or Rac1 silencing with siRNA fragment attenuated Rab23 promoted cells migration and invasion. Notably, we confirmed that Rab23 was co-localized with integrin β1 in cell membrane of Rab23 WT and Rab23 Q68L stable expression cells and Rab23 efficiently coprecipitated with integrin β1 and Tiam1 in a GTP-dependent manner. Further, integrin β1 siRNA interrupted the coprecipitation between Rab23 and Tiam1 and attenuated Rab23 promoted cells migration and invasion. Taken together, our results indicated that Rab23 promotes squamous cell carcinoma cells migration and invasion by regulating Integrin β1/Tiam1/Rac1 pathway.

## INTRODUCTION

Cutaneous squamous cell carcinoma (cSCC) is the second most common form of non-melanoma skin cancer. The incidence of SCC is about 10,000 per year in England and Wales, 700,000 per year in the US, resulting in approximately 2,500 deaths. The incidence is higher in Caucasians. The five-year rate of recurrence of primary cutaneous lesions is 8 percent, and the five-year rate of metastasis is 5 percent. The fatality rate of metastasis cSCC is about 40 percent [1]. The majority of cSCC cases are readily treatable by surgery, radiotherapy, photodynamic therapy, and drugs with a good chance of achieving cure [2, 3, 4]. However, in a subset of patients, cSCC can be biologically aggressive, showing a greater propensity for local recurrence and metastasis to regional lymph nodes and distant organs. The percentage of primary cSCCs that metastasize varies between case series but is usually under 5%. In high-risk cSCC, this percentage is higher [5], ranging from 15%(2) to 38%(1), depending on the study. Thus, an urgent better understanding of precise molecular mechanisms of metastasis cSCC and more efficacy therapeutic target are needed for improving clinical outcome of this fatal disease.

Rab23, a member of Ras-related small GTPase family, was isolated in the brain in 1994. In 2001, Eggenschwiler et al. firstly demonstrated that Rab23 is a cell autonomous negative regulator of the mouse Shh signaling pathway in open brain mouse mutants, suggesting that Rab23 plays a key role in the regulation of Shh signaling pathway [6–9]. Genetic evidence indicates that Rab23 regulates downstream of Sonic hedgehog (Shh), SMO and PTCH1, and possibly upstream or at the level of Gli transcriptional factors. Recently, Chi et al reports that Rab23 negatively regulates Gli1 transcriptional factor in a Su(Fu)-dependent manner [10]. However, in a study on chondrocyte differentiation, down-regulation of Rab23 was shown to decrease the level of Gli1 in chondrocytes, suggestive of a positive role of Rab23 in Gli1 regulation [11]. In addition, there are evidences that Rab23 plays a negative regulator in some carcinogenesis, such as thyroid cancer [12]. Down-regulation of Rab23 was seen in three thyroid malignant cohorts; follicular thyroid carcinoma, papillary thyroid carcinoma and follicular variant of papillary thyroid carcinoma when compared with the benign follicular adenoma group [12]. However, some researchers recognized it as an oncogene recently. In HCC, Rab23 was found high cytoplasmic and nuclear expression, which is correlated with tumor size. Knocking down Rab23 suppressed Hep3B cell growth [13]. In many lung cancer specimens, Rab23 was found localized in the nuclei, but no Rab23 expression was detected in normal lung tissue. In gastric cancers, researchers identified Rab23 as an invasion mediator gene in diffuse-type gastric cancer by using integrative genomics and further demonstrated that overexpression of Rab23 can enhance cell invasion of gastric cancer cells through cell invasion assay *in vitro* [14].

In the present study, we investigate the expression of Rab23 in AKs, SCC in situ, invasive SCC and normal skin by immunohistochemistry, and we found Rab23 was upregulated in SCC tissues and cell lines, which strongly promoted migration and invasion of SCC cells by the activating of Rac1 GTPase. Moreover, we found a direct interaction among integrin β1, Rab23 and Tiam1, the direct interaction between Rab23 and Tiam1 was disappeared after integrin β1 siRNA.

## RESULTS

### Rab23 was upregulated in AK, SCC in situ and invasive SCC

**Figure 1:**
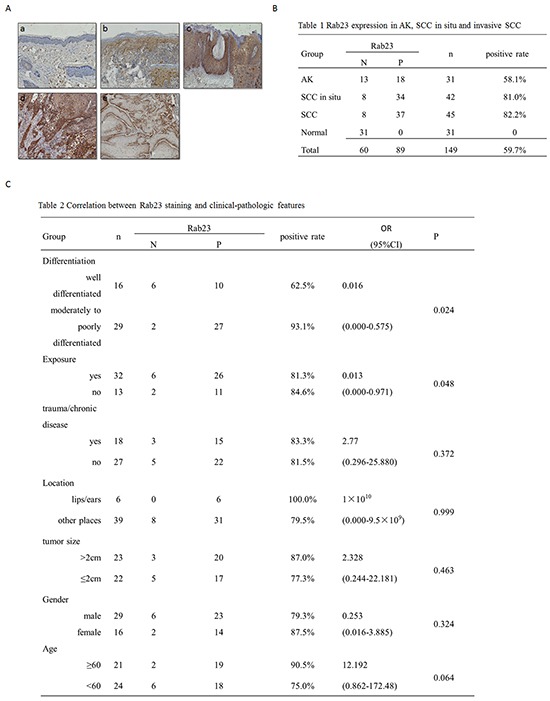
Rab23 was upregulated in AK, SCC in situ and invasive SCC **A.** The immunohistochemistry assay of Rab23 expression in AK, SCC in situ and invasive SCC. **a.** Rab23 proteins were absent in normal epidermis or dermis. **b.** Rab23 was distributed in basal layer and lower spinous layer in AK lesion. **c.** Positively stained cells were distributed in the full-thickness epidermissquamous atypia of SCC in situ. **d.** Strong staining was observed in the moderately to poorly differentiated tumors. **e.** Rab23 staining was observed at the base of well-differentiated tumor. No staining was observed in the well-differentiated keratinocytes or keratin pearls in well-differentiated tumors. **B.** Rab23 expression in AK, SCC in situ and invasive SCC. **C.** Correlation between Rab23 staining and clinical-pathologic features.

In 31 normal skin sections, Rab23 protein was absent in normal epidermis or dermis (Figure 1Aa). In 31 AK lesions, Rab23 was distributed in basal layer or lower spinous layer, but not in upper spinous layer, granular layer, horny layer or dermis (Figure 1Ab). Positively stained cells were distributed in the full-thickness epidermis squamous atypia of SCC in situ (Figure 1Ac). In biopsies of invasive squamous cell carcinoma, expression of Rab23 greatly varied depending on the level of tumor differentiation. Strong staining was observed in the moderately to poorly differentiated tumors (Figure 1Ad) or at the base of well-differentiated tumor (Figure 1Ae). No staining was observed in the well-differentiated keratinocytes or keratin pearls in well-differentiated tumors (Figure 1Ae). Strong cytoplasmic and cytomembrane staining was seen. The results of immunohistochemistry in AK, SCC in situ, invasive SCC and normal skin were summarized in Figure 1B. Rab23 expression was more frequent in SCC in situ and invasive SCC than AK.

The relationship between Rab23 and clinical-pathological characteristics was analyzed by logistic regression analysis. In 45 invasive SCC sections, moderately to poorly tumor differentiation and nonexposed positions is the risk factors of Rab23 positive staining. There was no statistically significant difference in Rab23 expression according to trauma/chronic disease, location on lips/ears, tumor size, gender, or age (Figure 1C).

### Rab23 promotes squamous cell carcinoma cells migration and invasion

**Figure 2:**
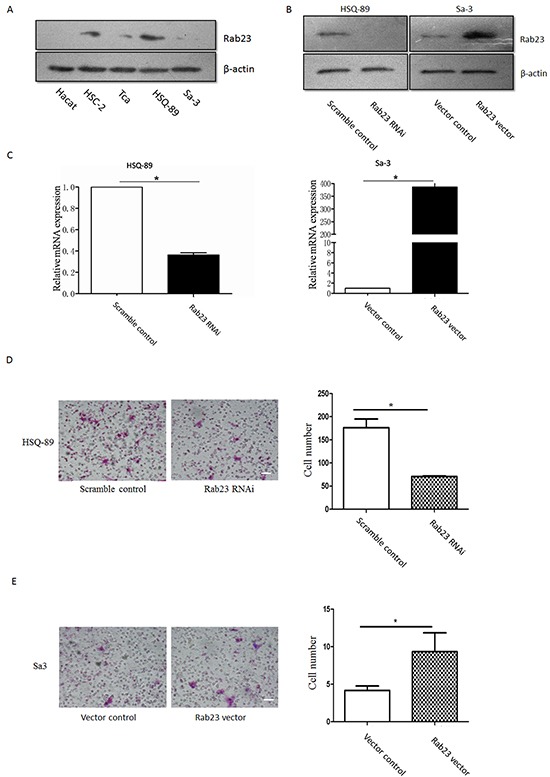
Rab23 promoted squamous cell carcinoma cells migration and invasion **A.** Hact, HSC-2, Tca, HSQ-89 and Sa3 cells were lysed, western blotting was used to detected the protein expression of Rab23. HSQ-89 cells were transfected with scramble or Rab23 siRNA, Sa3 cells were transfected with control vector or Rab23 overexpression vector, then the expression of Rab23 protein was detected by western blotting **B.**, the expression of Rab23 mRNA was detected by qPCR **C. D.** Invasive migration of HSQ-89 cells transfected with scramble or Rab23 siRNA (left), Quantification of the Transwell invasion assay (right), data is presented as the mean ± SEM; *P* values were calculated using the Student's *t*-test, *P < 0.05. **E.** Invasive migration of Sa3 cells transfected with control vector or Rab23 overexpression vector (left), Quantification of the Transwell invasion assay (right), data is presented as the mean ± SEM; *P* values were calculated using the Student's *t*-test, *P < 0.05.

To identify the function of Rab23 in SCC, we firstly examined Rab23 expression in SCC cell lines and HaCaT Keratinocytes, western blot analysis revealed that Rab23 was upregulated in all SCC cell lines, the most abundant Rab23 expression were in HSC-2 and HSQ-89, the less abundant Rab23 expression were in Tca and Sa3 (Figure 2A). Transient transfected HSQ-89 or Sa3cell with Rab23 siRNA or Rab23 vector decreased or increased Rab23 mRNA or protein expression (Figure 2B, 2C). Silencing of Rab23 with siRNA fragment suppressed cell invasion in HSQ-89 cell line, while overexpression of Rab23 increased Rab23 mRNA and protein expression and promoted cell invasion in Sa3 cell lines (Figure 2D, 2E).

### Rab23 promotes squamous cell carcinoma cells migration and invasion in GTP-bound form of Rab23

**Figure 3:**
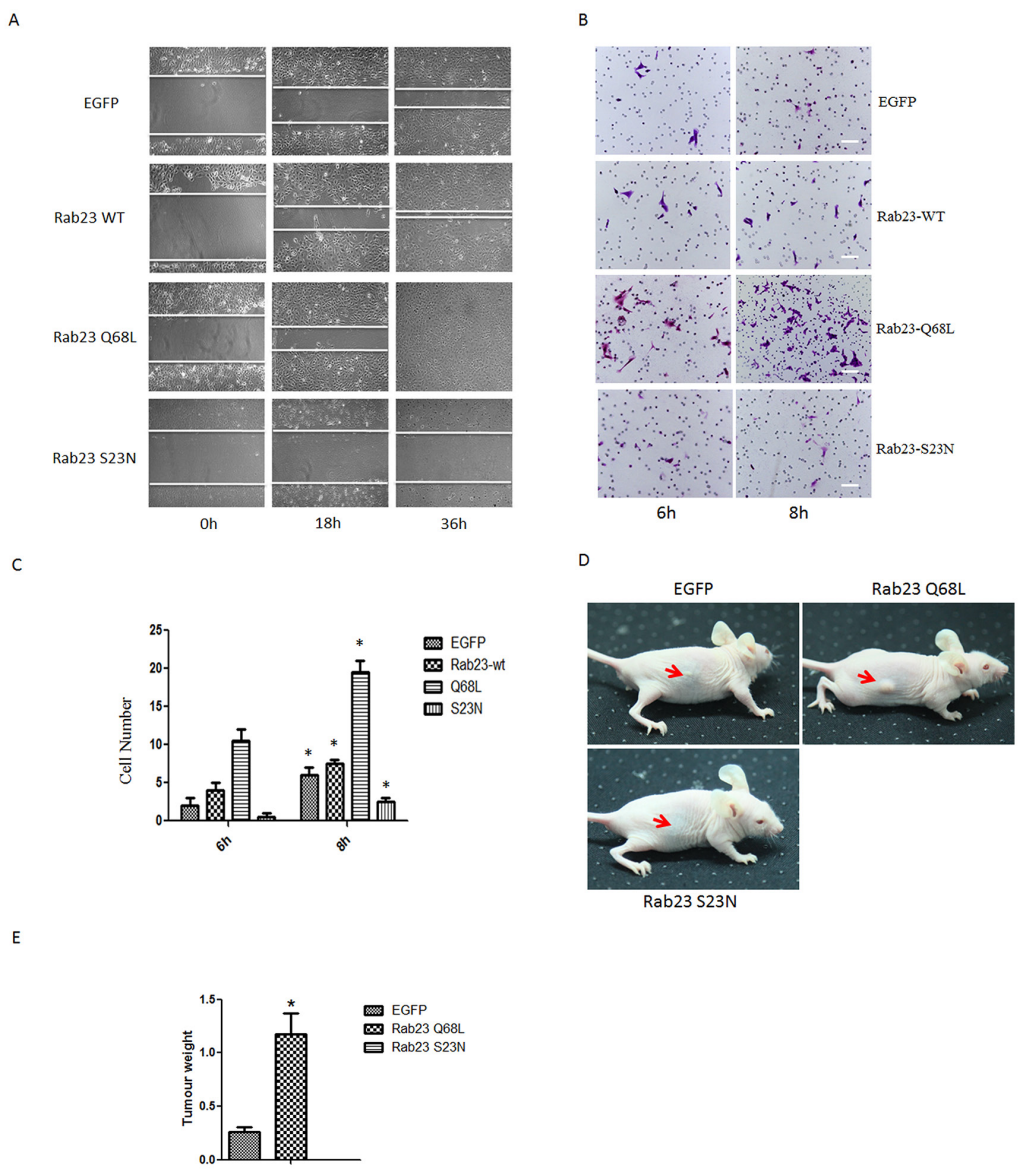
Rab23 promotes squamous cell carcinoma cells migration and invasion in GTP-bound form of Rab23 **A.** Representative images of the wound healing assay with Sa3 cells expressing EGFP, Rab23 WT, Rab23 Q68L and Rab23 S23N. **B.** Invasive migration of Sa3 cells expressing EGFP, Rab23 WT, Rab23 Q68L and Rab23 S23N. Bar = 200 μm **C.** Quantification of the Transwell invasion assay, data is presented as the mean ± SEM; *P* values were calculated using the Student's *t*-test, *P < 0.05. **D.** The representative images for tumor formation in nude mice with Sa3 cells expressing EGFP, Rab23 Q68L and Rab23 S23N. **E.** The tumour weight assay of tumor formation in nude mice, data is presented as the mean ± SEM; *P* values were calculated using the Student's *t*-test, *P < 0.05.

Furthermore, to determine whether Rab23 promotes cell invasion resulted from GTP-bound form of Rab23, we overexpressed Rab23 Q68L or Rab23 S23N that are constitutively GTP or GDP bound forms and act in a dominant active or dominant negative manner respectively. As results, overexpression of Rab23 wild-type (WT) or Rab23 Q68L promoted cell migration and invasion, but overexpression of Rab23 S23N reduced cell migration and invasion (Figure 3A–3C). In order to confirm the finding in cells, tumor formation assay was done in nude mice. As shown in Figure 3D and 3E, Rab23 Q68L promoted tumor formation while Rab23 S23N restrained tumor formation, compared to control (EGFP). These results indicated that Rab23 promotes squamous cell carcinoma cells migration and invasion in GTP-bound form of Rab23.

### Rab23-promoted squamous cell carcinoma cells migration and invasion requires activation of Rac1

**Figure 4:**
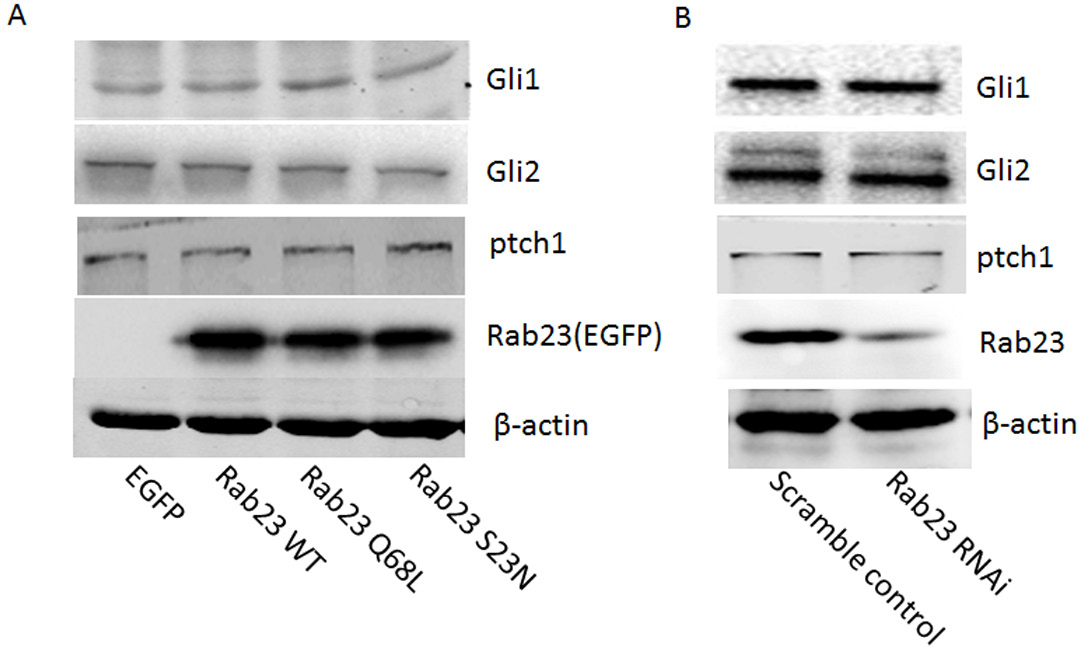
Rab23 didn't promote squamous cell carcinoma cells migration and invasion via Shh pathway **A.** Overexpression of Rab23 didn't inhibit the expression of ptch1, Gli1 and Gli2. **B.** Downregulation of Rab23 didn't promote the expression of ptch1, Gli1 and Gli2.

**Figure 5:**
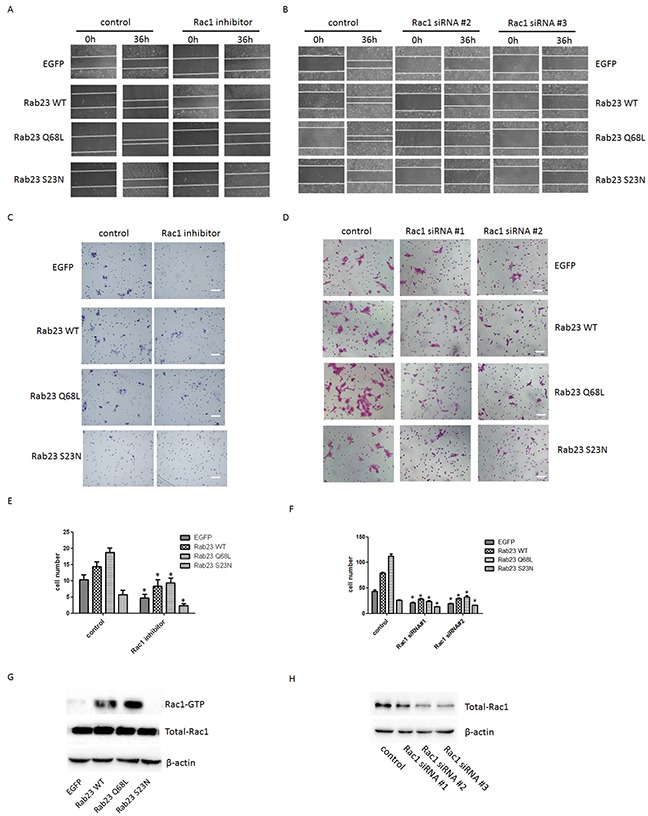
Rab23-promoted squamous cell carcinoma cells migration and invasion requires activation of Rac1 **A.** Representative images of the wound healing assay with Sa3 cells expressing EGFP, Rab23 WT, Rab23 Q68L and Rab23 S23N after Rac1 inhibitor treatment. **B.** Representative images of the wound healing assay with Sa3 cells expressing EGFP, Rab23 WT, Rab23 Q68L and Rab23 S23N after Rac1 siRNA transfection. **C.** Invasive migration of Sa3 cells expressing EGFP, Rab23 WT, Rab23 Q68L and Rab23 S23N after Rac1 inhibitor treatment. Bar = 100 μm. **D.** Invasive migration of Sa3 cells expressing EGFP, Rab23 WT, Rab23 Q68L and Rab23 S23N after Rac1 siRNA transfection. Bar = 200 μm. **E.** Quantification of the Transwell invasion assay in C, data is presented as the mean ± SEM; *P* values were calculated using the Student's *t*-test, *P < 0.05. **F.** Quantification of the Transwell invasion assay in D, data is presented as the mean ± SEM; *P* values were calculated using the Student's *t*-test, *P < 0.05. **G.** Levels of active GTP-bound Rac1in Sa3cells expressing EGFP, Rab23 WT, Rab23 Q68L and Rab23 S23N were detected by GSTpull down assays, as described in the materials and methods. Total Rac1 present in the lysates were analysed by immunoblotting with anti-Rac1 antibody. **H.** Quantification of Rac1 protein expression by western blotting in Sa3 cells transfected with Rac1 siRNA.

To investigate the molecular mechanisms that Rab23 promotes squamous cell carcinoma cells migration and invasion, the expression level of key molecule in Shh signaling pathway, ptch1, Gli1 and Gli2, were detected in our experiment, as Rab23 acts as a negative regulator of the Shh signaling pathway. The results showed that Rab23 upregulation didn't inhibit the expression of ptch1, Gli1 and Gli2 (Figure 4A). Rab23 downregulation didn't promote the expression of ptch1, Gli1 and Gli2 (Figure 4B). These results suggested that Rab23 didn't promote squamous cell carcinoma cells migration and invasion via Shh pathway. Further, GST-pull down assay was performed to detect the active GTP-bound the activity of Rac1, as RAC1 are known to promote cell invasion and motility through different mechanisms. As shown in Figure 5G, the active conformation of GTP-bound Rac1 was upregulated in Rab23 WT and Rab23 Q68L stably expressing cells, while GTP-bound Rac1 was downregulated in Rab23 S23N stably expressing cells. The results indicated that Rac1 activation was involved in Rab23 promoted squamous cell carcinoma cells migration and invasion. Thus, Rac1 inhibitor was used to inhibit the activation of Rac1, wound migration assay and an *in vitro* invasion assay was performed to investigate the cell migration and invasion. The results shown that Rac1 inhibitor significantly attenuated Rab23 promoted cell migration and invasion (Figure 5A, 5C, 5E). In order to confirm this, we knocked down Rac1 with Rac1 siRNA (Figure 5H) and found that cell migration and invasion promoted by Rab23 were reduced after Rac1 siRNA transfetion (Figure 5B, 5D, 5F).

### Rab23 regulates Rac1 by directly binding with integrin-β1 in a GTP-dependent manner

It was reported that colocalization of Rab5 and Rab23 was visible in cells [7] and Rab5 was involved in recruiting integrin β1 which was proved to be required for the activation of Rac1[15], a member of the Rho GTPases subfamily, and involve in the regulation of actin cytoskeletal organization via Tiam1[16]. Thus, Co-IP assay was used to investigate the interaction among Rab23, Rab5, integrin β1 and Tiam1. We found that Rab23 efficiently coprecipitated with integrin β1 and Tiam1 in a GTP-dependent manner, but not with Rab5 (Figure 6B). Further, we observed that Rab23 was colocalized with integrin β1 in cell membrane of Rab23 WT and Rab23 Q68L stable expression Sa3 cells (Figure 6A). These results indicated that Rab23 coprecipitated with integrin β1 and Tiam1 and recruit integrin β1 in a GTP-dependent manner.

**Figgure 6:**
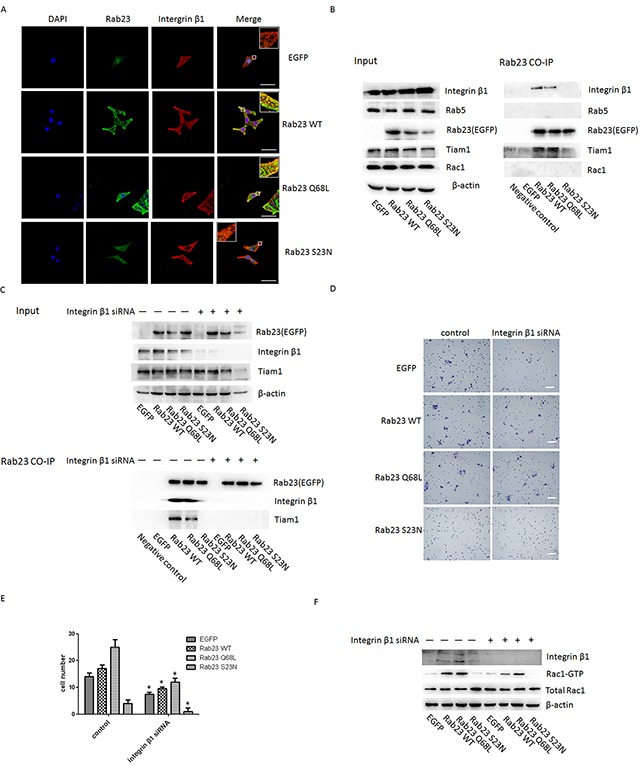
Rab23 regulates Rac1 by directly associating with integrin-β1 in a GTP- dependent manner **A.** Sa3 cells expressing EGFP, Rab23 WT, Rab23 Q68L and Rab23 S23N were stained with integrinβ1 (red) and DAPI (blue), respectively. Bar = 50 μm. **B.** Sa3 cells expressing EGFP-Rab23 WT, EGFP-Rab23 Q68L, EGFP-Rab23 Q68L, or EGFP alone were lysed, and these fusion proteins immunoprecipitated with ananti-EGFP antibody. The resulting immunoprecipitates were screened for the presence of integrin β1, Rab5, Tiam1and Rac1 by immunoblotting for their specific antibody respectively. **C.** Sa3 cells expressing EGFP-Rab23 WT, EGFP-Rab23 Q68L, EGFP-Rab23 Q68L, or EGFP alone after integrinβ1 siRNA were lysed, and these fusion proteins immunoprecipitated with an anti-EGFP antibody. The resulting immunoprecipitates were screened for the presence of integrin β1 and Tiam1 by immunoblotting for their specific antibody respectively. **D.** Invasive migration of Sa3 cells expressing EGFP, Rab23 WT, Rab23 Q68L and Rab23 S23N after integrinβ1 siRNA transfection. Bar = 100 μm. **E.** Quantification of the Transwell invasion assay in D, data is presented as the mean ± SEM; *P* values were calculated using the Student's *t*-test, *P < 0.05. **F.** Quantification of Rac1-GTP protein expression by western blotting in Sa3 cells transfected with integrin β1 siRNA.

Next, we knocked down integrin β1 with integrinβ1 specific siRNA. As shown in Figure 6C, Rab23 didn't coprecipitate with Tiam1 after integrinβ1 siRNA. The result revealed that co-precipitation between rab23 and Tiam1 is dependent on integrin β1. Moreover, cell invasion and activation of Rac1 was significantly suppressed after integrinβ1 siRNA (Figure 6D, 6E, 6F).

## DISCUSSION

Here, we have detailed that Rab23 was upregulated in SCC tissues and cell lines, which strongly promoted migration and invasion of SCC cells, and we found this appears to be mediated by the activation of Rac1 GTPase. Further, we revealed a direct interaction among integrin β1, Rab23 and Tiam1, the direct interaction between Rab23 and Tiam1 was disappeared after integrin β1 siRNA.

RAB23, a Rab-GTPase vesicular transport protein, is mutated in open brain mice [6]. It appears to antagonize sonic hedgehog (Shh)-mediated signaling during mouse development [17]. Rab23 acts on downstream of Smo and upstream of Gli transcription factors in patterning neural cell types in the spinal cord [8]. As SHH signaling has been shown to be tumorigenic in various cancer types such as small cell lung cancer, colorectal adenocarcinoma, basal cell carcinoma, and even SCC itself, Rab23 was recognized as a negative regulator of carcinoma. In thyroid cancer, Rab23 was downregulated compared with the benign follicular adenoma [12]. But in HCC, lung cancer, and gastric cancers, Rab23 was found high expression [18, 12-14]. Some researchers recognized it as an oncogene recently [19]. In our study, Rab23 expression was altered during cSCC development. It seemed that Rab23 expression was associated with cellular atypia. In biopsies of invasive SCC, expression of Rab23 greatly varied depending on the level of tumor differentiation. Strong staining was observed in most moderately to poorly differentiated tumors, particularly in anaplastic cells, whereas most well-differentiated tumors were negative staining (Figure 1Ad, 1Ae). Besides, Rab23 was more frequent in SCC in situ and invasive SCC than AK (Figure 1Ab, 1Ac, 1Ad). It seemed Rab23 represent a potential invasive ability and contributed to tumorigenesis in cSCC. Logistic regression analysis between Rab23 and clinical-pathological characteristics showed that moderate to poor differentiation and nonexposed positions were the risk factors of Rab23 positive staining (Figure 1C). These two factors were also the risk factor of metastasis. The statistic analysis shows that Rab23 expression is related to some risk factor of metastasis, which hinted that Rab23 was involved in the invasion of cSCC.

Rac1, a small G-proteins widely implicated in tumorigenesis and metastasis, which transduced signals from tyrosine-kinase, G-protein-coupled receptors (GPCRs), and integrins, and controlled a number of essential cellular functions including motility, adhesion, and proliferation[20]. The activation of Rac1 activation in tumors might play a role in cancer progression. Once activated, Rac1 could interact with numerous downstream effectors, with the resulting modulation of a variety of different signaling pathways and cellular functions [21–26]. We found that Rab23 WT and Rab23 Q68L overexpression promoted cell migration and invasion and Rac1 was activated in Rab23 WT and Rab23 Q68L overexpression Sa3 cells (Figure 5G). However, cell migration and invasion promoted by Rab23 were reduced after Rac1 inhibition or knock down (Figure 5A–5D). The results indicated that Rab23 promotes squamous cell carcinoma cells migration and invasion requires activation of Rac1.

Futher, we investigate the mechanism Rab23 activated Rac1. It was reported that colocalization of Rab5 and Rab23 was visible in cells[8] and Rab5 was involved in recruiting integrin β1 which was proved to be required for the activation of Rac1[16]. And Tiam1, a specific GEF of Rac1 was reported to be recruited to integrin β1 complexes by 14-3-3ζ where it mediates integrin-induced Rac1 activation and motility[17]. Thus, the Co-IP assay was used to investigate the interreaction among Rab23, Rab5, integrin β1 and Tiam1. The results shown Rab23 efficiently coprecipitated with integrin β1 and Tiam1 in a GTP-dependent manner and Rab23 was colocalized with integrin β1 in cell membrane of Rab23 WT and Rab23 Q68L stable expression Sa3 cells (Figure 6B, 6A). Besides, we found Rab23 could not coprecipitated with Tiam1 after integrinβ1 knock down (Figure 6C) and cell invasion promoted by Rab23 were reduced after integrinβ1 knock down (Figure 6D, 6E). The results indicated that Rab23 recruit integrinβ1 in a GTP-dependent manner, then integrinβ1 recruited Tiam1 and activated Rac1.

In conclusion, our results demonstrated that Rab23 was upregulated in SCC tissues and cell lines, which strongly promoted migration and invasion of SCC cells by activating Rac1. Further, we revealed a direct interaction among integrin β1, Rab23 and Tiam1, the direct interaction between Rab23 and Tiam1 was disappeared after integrin β1 knock down.

## MATERIALS AND METHODS

### Antibodies and DNA constructs

Commercial antibodies against the following antigens were used: Rab23 (Proteintech, Chicago, USA), integrin-β1(Abcam, Cambridge, UK), Rac1(millipore, Billerica, USA), Tiam1 (Abcam, Cambridge, UK), Rab5(Abcam, Cambridge, UK), GFP (Santa Cruz, Dallas, USA), and fluorescently conjugated secondary antibodies (cwbiotech, Peking, China). Full-length human Rab23 was cloned into a pcDNA 3.1/V5-His vector as a gift from professor xie (Indiana University School of Medicine, Indiana, USA)

### Patient material

A total of 149 tissue samples were selected from the pathologic database and tissue archives of the dermatology department, Xijing hospital, the Forth Military medical University. Thirty-one specimens had been diagnosed as actinic keratosis(M/F: 22/9; 24-87 years, midean: 60), forty-two as Bowen's disease or SCC in situ(M/F: 27/15; 40-86 years, midean: 64), forty-five SCC(M/F: 29/11; 37-88 years, midean: 59) and thirty-one normal skin(M/F: 21/10; 22-87 years, median: 56) as control. The ages and gender of the lesions were anticipated to show a similar distribution with each group.

### Immunohistochemistry

Tissues were first sectioned at 4 μm. The sections were deparaffinized in xylene and hydrated in a graded ethanol series to distilled water. The slides were pretreated in citrate buffer (pH 6.0) in a microwave oven for 20 min (steaming for 2.5 min). Sections were rinsed in distilled water and treated with 3% H_2_O_2_ for 10 min. Slides were rinsed in Phosphate Buffered Saline (PBS) and incubated in the Rab23 (Product description, Chicago, USA) primary antibody overnight at a dilution of 1 : 100. Slides were rinsed in PBS buffer and incubated with a secondary reagent (ElivisionTM plus Polyer HRP (Mouse/Rabbit) IHC Kit) according to the manufacturer's (Maixin, Fuzhou, China). The peroxidase reaction was visualized using diaminobenzidine (DAB). Scoring was done by two independent observers blinded to the clinical detail. For analysis of the Rab23 in the four groups, statistical analysis was performed using χ2 testing or Fisher exact testing when frequencies were smaller than five. Logistic regression analysis was used to analyse the relationship between Rab23 and clinical and pathological characteristics by SPSS 11.5. P < 0.05 was considered significant.

### Cell lines and siRNA transfections

Sa3 and HSQ-89 squamous cell carcinoma cells (American Type Culture Collection) were grown in Dulbecco's Modified Eagle's Minimal Essential Medium (DMEM) (Gibco-Invitrogen) supplemented with 10% fetal bovine serum (Gibco-Invitrogen) in a humidified atmosphere containing 5% CO2 at 37°C. siRNAs targeting Rab23 (sense, 5′-caaacaaaggaccaagaaaTT-3′ and 5′-uuucuugguccuuuguuugTT-3′, genepharma), or scramble control siRNA (sense, 5′-uucuccgaacgugucacguTT-3′ and 5′-acgugacacguucggagaaTT-3′, genepharma) were transfected at 100nM concentration to cells using Lipofectamine 2000 (Invitrogen) according to the manufacturer's instructions. siRNAs targeting Rac1 and Integrin β1 were transfected at 100nM concentration to EGFP, Rab23 WT, Rab23 Q68L or Rab23 S23N stable expression cells using Lipofectamine 2000 (Invitrogen) according to the manufacturer's instructions.

### GST-pull down assay of GTP-bound Rac1

GST-pull down assays of active GTP-bound Rac1was performed as protocol with instructions. Cells were lysed in lysis buffer. Equal amount of cell lysates were incubated with GST-RBD beads for 60 min at 4°C. GTP-bound Rac1 was detected by Western blotting. The amount of GTP-bound Rac1 was normalized to the total amount of these GTP-bound GTPases in cell lysates in each sample separately.

### *In vivo* analysis

Sa3 cells expressing EGFP, Rab23 Q68L and Rab23 S23N were grown in Dulbecco's Modified Eagle's Minimal Essential Medium (DMEM) (Gibco-Invitrogen) supplemented with 10% fetal bovine serum (Gibco- Invitrogen) in a humidified atmosphere containing 5% CO2 at 37°C. Then nude female mice at 6 weeks of age were injected with 1.0 × 10^6^ cells form tail vein. Tumor formation was thereafter imaged every two days.

### Lentivirus generation and establishment of Rab23 stable expression and Rab23 RNAi cells

Full-length human Rab23 cDNA was cloned into the Nhe I/Nhe I sites of the pGC-FU-EGFP-NSC-IRES-Puromycin vector, which tags the N-terminal end of Rab23 protein with a EGFP epitope. Lentiviral shRNA construct was obtained by annealing the predesigned primers and subsequent cloning into the AgeI-EcoRI sites of the GV248 lentiviral vector that also co-expressed green fluorescent protein as a selection marker. Primers targeting nucleotides 668 -686 of human Rab23 mRNA were 5′- CcggTCAATCTTAGA- CCCAACAA CTCGAG TTGTTGGGTCTAAGATTGATTTTTg-3′ (sense) and 5′-aattcaaaaa TCAATCTTAGACCCAACAACTCGAG TTGTTGGGTCTAAGA- TTGA-3′ (antisense). For generation of lentivirus, 293T packaging cells, were seeded at a density of 3 × 10^6^ cells on 10-cm culture plates. After 24 h, successful co-transfection of plasmid including the Scramble RNA, Rab23 RNAi, EGFP, Rab23 WT, Rab23 Q68L or Rab23 S23N with pHelper 1.0 and pHelper 2.0 plasmid by the Lipofectamine 2000 method according to the manufacturer's instructions. After 48 h, supernatant was collected and added to PEG-*it*™ virus precipitation solution. The supernatant/PEG-*IT* mixture was centrifuged 1,500 × *g* for 30 min at 4°C and then resuspended in DMEM medium at 1/100 of the original volume. One day prior to transduction, Sa3 cells were plated in 6-well plates at 5 × 10^4^ cells. Then, Sa3 cells were infected with lentiviral particles containing Scramble RNA, Rab23 RNAi, EGFP, Rab23 WT, Rab23 Q68L or Rab23 S23N for 8h. The next day, the medium was removed and Sa3 cells were cultured in DMEM medium including puromycin (2 μg/ml) for 72h. The transduction efficiency of Sa3 cells with EGFP signals were determined by Inverted fluorescence microscope. The expanded cells were then used for further experiments.

### Immunofluorescence

Sa3 cells were plated onto acid-washed glass coverslips. Cells were fixed with 4% PFA, permeabilized with 0.1% Triton X-100 and 2% BSA in PBS for 20 min, and then blocked with 2% BSA in PBS. Primary antibodies were used at 5–10 mg/ml and incubated overnight at 4°C. Cy3-conjugated secondary antibodies, at a concentration of 5 μg/ml, were incubated at RT for 1 h. Coverslips were mounted with 2% BSA in PBS containing DAPI to counterstain nuclei. Immunofluorescent samples were analyzed with an inverted confocal microscopy (Nikon).

### Immunoprecipitation and westernblot analysis

Cells were lysed using IP and westernblot lysis buffer. EGFP-tagged Rab23 was immunoprecipitated with anti-EGFP antibody (Santa Cruz, Dallas, USA) for 6 h, followed by incubation with A/G plus beads (Beyotime, Inc.) overnight. After washing the beads five times with IP and westernblot lysis buffer, the bound fractions were analyzed by 10% or 10% SDS-PAGE followed by immunoblotting with anti-EGFP rabbit antibody (1:500 dilution), anti-integrin β1 rabbit antibody (1:500 dilution), anti-Rab5 antibody (1:1000 dilution), anti-Tiam1 antibody (1:1000 dilution) and anti-Rac1 antibody (1:1000 dilution) Immunoreactive bands were visualized by enhanced chemiluminescence (ECL).

### Scratch-wound assay

Cells were planted on tissue-culture plastic, allowed to grow to a density of 90%, and serum-starved overnight. Wounds were applied with a pipette tip and washed thoroughly with PBS. Wound closure was thereafter imaged in serum-free medium for the indicated times using an inverted widefield microscope (Nikon). Wound closure efficiency was calculated as percentage of wound area after the imaging (indicated times) compared with imaging starting time. Cell motility was analyzed in more detail by determining the displacement of cells over time and calculating migration speed and directionality as described for the migration assay.

### Invasion assay

The cell invasion assay was performed with matrigel (BD Biosciences, Sparks, MD) coated on the upper surface of a Transwell chamber (BD Falcon). The cells that had invaded through the membrane were fixed with 4% paraformaldehyde and stained with coomassie brilliant blue methanol. Photographs of 3 randomly selected fields of the fixed cells were taken, and the cells were counted.

## References

[1] Jemal A, Siegel R, Ward E, Hao Y, Xu J, Murray T, Thun MJ (2008). Cancer statistics. CA Cancer J Clin,.

[2] Clayman GL, Lee JJ, Holsinger FC, Zhou X, Duvic M, El-Naggar AK, Prieto VG, Altamirano E, Tucker SL, Strom SS, Kripke ML, Lippman SM (2005). Mortality risk from squamous cell skin cancer. J Clin Oncol.

[3] Buethe D, Warner C, Miedler J, Cockerell CJ (2011). Focus issue on squamous cell carcinoma: practical concerns regarding the 7th edition AJCC staging guidelines. J Skin Cancer.

[4] Lansbury L, Leonardi-Bee J, Perkins W, Goodacre T, BathHetxall FJ, Tweed JA (2010). Interventions for non-metastatic squamous cell carcinoma of the skin. Cochrane Database Syst Rev.

[5] Oddone N, Morgan GJ, Palme CE, Perera L, Shannon J, Wong E, Gebski V, Veness MJ (2009). Metastatic cutaneous squamous cell carcinoma of the head and neck: the Immunosuppression, Treatment Extra-nodal spread, and Margin status (ITEM) prognostic score topredict outcome and the need to improve survival. Cancer.

[6] Eggenschwiler JT, Espinoza E, Anderson KV (2001). Rab23 is an essentia negative regulator of the mouse Sonic hedgehog signalling pathway. Nature.

[7] Evans TM, Ferguson C, Wainwright BJ, Parton RG, Wicking C (2003). Rab23, a negative regulator of hedgehog signaling, localizes to the plasma membrane and the endocytic pathway. Traffic,.

[8] Eggenschwiler JT, Bulgakov OV, Qin J, Li T, Anderson KV (2006). Mouse Rab23 regulates hedgehog signaling from smoothened to Gli proteins. Dev Biol.

[9] Boehlke C, Bashkurov M, Buescher A, Krick T, John AK, Nitschke R, Walz G, Kuehn EW (2010). Differential role of Rab proteins in ciliary trafficking: Rab23 regulates Smoothened levels. J Cell Sci.

[10] Chi S, Xie G, Liu H, Chen K, Zhang X, Li C, Xie J (2012). Rab23 negatively regulates Gli1 transcriptional factor in a Su(Fu)-dependent manner. Cell Signal.

[11] Yang L, Clinton JM, Blackburn ML, Zhang Q, Zou J, Zielinska-Kwiatkowska A, Tang BL, Chansky HA (2008). Rab23 regulates differentiation of ATDC5 chondroprogenitor cells. J Biol Chem.

[12] Denning KM, Smyth PC, Cahill SF, Finn SP, Conlon E, Li J, Flavin RJ, Aherne ST, Guenther SM, Ferlinz A, O'Leary JJ, Sheils OM (2007). A molecular expression signature distinguishing follicular lesions in thyroid carcinoma using preamplification RT-PCR in archival samples. Mod Pathol.

[13] Liu YJ, Wang Q, Li W, Huang XH, Zhen MC, Huang SH, Chen LZ, Xue L, Zhang HW (2007). Rab23 is a potential biological target for treating hepatocellular carcinoma. World J Gastroenterol.

[14] Hou Q, Wu YH, Grabsch H, Zhu Y, Leong SH, Ganesan K, Cross D, Tan LK, Tao J, Gopalakrishnan V, Tang BL, Kon OL, Tan P (2008). Integrative genomics identifies RAB23 as an invasion mediator gene in diffuse-type gastric cancer. Cancer Res.

[15] Liu SS, Chen XM, Zheng HX, Shi SL, Li Y (2011). Knockdown of Rab5a expression decreases cancer cell motility and invasion through integrin-mediated signaling pathway. J Biomed Sci.

[16] O'Toole TE, Bialkowska K, Li X, Fox JE (2011). Tiam1 is recruited to β1-integrin complexes by 14-3-3ζ where it mediates integrin-induced Rac1 activation and motility. J Cell Physiol.

[17] Wang Y, Ng EL, Tang BL (2006). Rab23: what exactly does it traffic?. Traffic.

[18] Huang S, Yang L, An Y, Ma X, Zhang C, Xie G, Chen ZY, Xie J, Zhang H (2011). Expression of hedgehog signaling molecules in lung cancer. Acta Histochem,.

[19] Wang G, Cui Y, Zhang G, Garen A, Song X (2009). Regulation of proto-oncogene transcription, cell proliferation, and tumorigenesis in mice by PSF protein and a VL30 noncoding RNA. Proc Natl Acad Sci U S A.

[20] Wertheimer E, Gutierrez-Uzquiza A, Rosemblit C, Lopez-Haber C, Sosa MS, Kazanietz MG (2012). Rac signaling in breast cancer: a tale of GEFs and GAPs. Cell Signal.

[21] van Rijssel J, Hoogenboezem M, Wester L, Hordijk PL, Van Buul JD (2012). The N-terminal DH-PH domain of Trio induces cell spreading and migration by regulating lamellipodia dynamics in a Rac1-dependent fashion. PLoS One.

[22] Ungefroren H, Groth S, Sebens S, Lehnert H, Gieseler F, Fändrich F (2011). Differential roles of Smad2 and Smad3 in the regulation of TGF-β1-mediated growth inhibition and cell migration in pancreatic ductal adenocarcinoma cells: control by Rac1. Mol Cancer.

[23] Feng H, Hu B, Liu KW, Li Y, Lu X, Cheng T, Yiin JJ, Lu S, Keezer S, Fenton T, Furnari FB, Hamilton RL, Vuori K, Sarkaria JN, Nagane M, Nishikawa R, Cavenee WK, Cheng SY (2011). Activation of Rac1 by Src-dependent phosphorylation of Dock180 (Y1811) mediates PDGFRα-stimulated glioma tumorigenesis in mice and humans. J Clin Invest.

[24] Itoh RE, Kurokawa K, Ohba Y, Yoshizaki H, Mochizuki N, Matsuda M (2002). Activation of rac and cdc42 video imaged by fluorescent resonance energy transfer-based single-molecule probes in the membrane of living cells. Mol Cell Biol.

[25] Tang BL, Ng EL (2009). Rabs and cancer cell motility. Cell Motil Cytoskeleton.

[26] Rathinam R, Berrier A, Alahari SK (2011). Role of Rho GTPases and their regulators in cancer progression. Front Biosci (Landmark Ed).

